# High Speed Railway Environment Safety Evaluation Based on Measurement Attribute Recognition Model

**DOI:** 10.1155/2014/470758

**Published:** 2014-11-09

**Authors:** Qizhou Hu, Ningbo Gao, Bing Zhang

**Affiliations:** ^1^School of Automation, Nanjing University of Science & Technology, Nanjing, Jiangsu 2100984, China; ^2^East China Jiaotong University, Nanchang, Jiangxi 330013, China

## Abstract

In order to rationally evaluate the high speed railway operation safety level, the environmental safety evaluation index system of high speed railway should be well established by means of analyzing the impact mechanism of severe weather such as raining, thundering, lightning, earthquake, winding, and snowing. In addition to that, the attribute recognition will be identified to determine the similarity between samples and their corresponding attribute classes on the multidimensional space, which is on the basis of the Mahalanobis distance measurement function in terms of Mahalanobis distance with the characteristics of noncorrelation and nondimensionless influence. On top of the assumption, the high speed railway of China environment safety situation will be well elaborated by the suggested methods. The results from the detailed analysis show that the evaluation is basically matched up with the actual situation and could lay a scientific foundation for the high speed railway operation safety.

## 1. Introduction

According to the high speed railway safety operation research carried out in the laboratory of Nanjing University of Science and Technology, the high speed railway operation failure directly caused by bad environments accounts for 29% from July 2011 to December 2012, and comparatively the speed railway accidents in severe weather take up 81.4% of the total ones at the same time. The above statistics thus give us a better understanding of the fact that the bad weather has significant effects on the high speed railway safety operation.

In China, the current researches of environment impact on high speed railway can be mainly divided into the following two categories: first, the macrodisaster emergency prediction and warning system design and second, the microenvironmental factors impact mechanism analysis. As to the first one, Sun et al., Wang et al., and Tao et al. have outlined some key problems of high speed railway environment safety, such as alarm threshold, the layout of monitoring points, train controlling mode, and the basic component of high speed railway warning system [1–3]. Xiao et al., Calle-Sánchez et al., and Wang et al. also made an analysis of the potential factors which caused railway disaster from the following four aspects: personnel, equipment, management, and environment [4–6]. And Miyoshi and Givoni introduced analytic hierarchy process to set up railway environmental risk assessment system [7]. In the aspect of environmental factors impact mechanism, Zhou and Shen, Ling et al., and Lee et al. have made a specific discussion of such impact mechanism such as earthquake, wind, and other disasters in high speed railway from the view of engineering construction [8–10].

The comparison of the studies from abroad and home reveals that the researches of the high speed railway environment safety have been repeatedly carried out in an extremely earlier time and have been carefully studied by a lot of foreign researchers. Many countries have built up their own efficient high speed railway disaster warning system such as the Hokkaido and Shinkansen disaster warning system in Japan, which leads many other countries to conduct the earthquake prediction. For instance, France is now in possession of its Mediterranean earthquake monitoring system and Germany owns high speed railway disaster prevention system. Though the disaster monitoring systems of JingJingtang, Fuxia, and Wuguang have been already built in China, Zhang and Zeng contend that all the systems can be still well improved on the basis of the original ordinary railway disaster warning system [11] because there is a certain gap between foreign and China's high speed railway disaster warning systems after a relatively fair comparison.

Through the comparison of present researches between domestic and foreign, we can find that the domestic high speed railway disaster prevention is now in a transition from theory to practice, while foreign high speed railway disaster prevention system has been at a relatively perfect stage. Therefore, it is an urgent mission for the domestic researchers to make an intensive effort to the theory research of high speed railway disaster protection and system construction process so as to promote China high speed railway operating safety level.

## 2. High Speed Railway Environmental Impact Evaluation Indexes

### 2.1. High Speed Railway Index System of Environmental Impacts

The operational problems of the high speed railway are mainly caused by such uncertain factors as raining, thundering and lightning, horizontal wind, earthquake, and so forth, whose degree of intensity will directly decide the degree of danger posing to the high speed railway operation safety. The analysis of the characteristics of various environmental factors in the process of high speed railway operation in recent years and the conclusion of the mechanism of different environmental factors on high speed railway safe operation are presented in Table 1.

**Figure 1:**
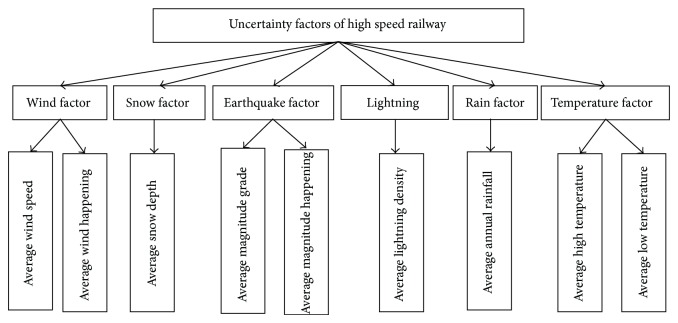
High speed railway environmental impact evaluation indexes system.

Besides the six factors listed in Table 1, problems in the high speed railway are also being influenced by debris flow and water and rock burst. However, given the complexity of geological conditions and the difficulty of data acquisition, we only use average annual rainfall, average annual maximum lightning density, annual disaster monsoon winds, average disasters incidence of monsoon, average magnitude grade, average incidence of earthquakes, average annual maximum snow depth, average highest temperature, and average minimum temperature as the environment factor evaluation index, which are shown in Figure 1.

It is necessary to be mentioned that the usual climate environment will not exert any influence upon the operation of high speed railway, except typhoon, sandstorm, blizzard, and earthquakes, while high or low temperatures have significant influence on the operation of high speed railway. Therefore, with the exclusive of the average rainfall in Figure 1, other factors represent the extreme climate environment. Each environmental factor evaluation index calculation formula and specification is shown in the following equations.

Average annual rainfall level is

(1) $$\begin{array}{c} \text{AAR}=\overset{N}{\underset{i=1}{\sum }}\frac{\text{R}{\text{F}}_{i}}{N}, \end{array}$$

where RF_*i*_ is the maximum rainfall in the *i*th  year (mm) and is the number of the years.

Maximum lightning density is

(2) $$\begin{array}{c} \text{MLD}=\overset{N}{\underset{j=1}{\sum }}\frac{\text{L}{\text{H}}_{j}}{\left(N\times \text{area}\right)}, \end{array}$$

where LH_*j*_ is the thunder lightning happening in certain region in the *j*th year (time) and area is the area of a city or region (m^2^).

Disasters wind speed is

(3) $$\begin{array}{c} \text{AWS}=\overset{W_{n}}{\underset{k=1}{\sum }}\frac{\text{DW}{\text{S}}_{k}}{W_{n}}, \end{array}$$

where DWS_*k*_ is the speed of the *k*th disaster wind (m/s) and area is the area of a city or region (Km^2^).

Average wind happening is

(4) $$\begin{array}{c} \text{AWH}=\frac{W_{n}}{N}, \end{array}$$

where *W*_*n*_ is the total times of the disaster wind happening (time).

Average magnitude grade is

(5) $$\begin{array}{c} \text{AMG}=\overset{D_{n}}{\underset{l=1}{\sum }}\frac{\text{M}{\text{G}}_{l}}{D_{n}}, \end{array}$$

where MG_*l*_ is the magnitude of the *l*th earthquake (degree) and *D*_*n*_ is the total times of the earthquake (time).

Average magnitude happening is

(6) $$\begin{array}{c} \text{AWH}=\frac{D_{n}}{N}. \end{array}$$

Average high and low temperature are

(7) $$\begin{array}{c} \text{AHT}=\overset{N}{\underset{p=1}{\sum }}\frac{\mathrm{Max}T_{p}}{N},\\ \text{ALT}=\overset{N}{\underset{p=1}{\sum }}\frac{\mathrm{Min}T_{p}}{N}, \end{array}$$

where *Max*⁡ *T*_*p*_ is the highest temperature in the *p*th year (°C) and *Min*⁡ *T*_*p*_ is the lowest temperature in the *p*th year (°C).

Average snow depth is

(8) $$\begin{array}{c} \text{ASD}=\overset{N}{\underset{r=1}{\sum }}\frac{\mathrm{Max}\;\text{S}{\text{D}}_{r}}{N}, \end{array}$$

where *Max*⁡ SD_*r*_ is the deepest depth in the *r*th year (cm).

### 2.2. Demarcation of the Environmental Climate Factor Affected Threshold (Modify)

#### 2.2.1. Threshold under Horizontal Wind Influence

The representative research about the effects of horizontal wind on high speed railway train running is conducted preciously in Japan, which calculates the horizontal wind velocity under the condition of critical capsize under different running speed by wind tunnel experiment and takes the critical wind speed as the threshold of Shinkansen disaster warning (Table 2). China's high speed railway line train CRH series are characterized by the similar features with those of Japanese train in the shape and the axle load. Therefore, the Japanese Shinkansen warning horizontal wind speed is adopted as the influencing factors of high speed railway in our country horizontal wind threshold.

#### 2.2.2. Threshold under Earthquake Influence

In terms of the research results at home and abroad, the calculation of earthquake alarm threshold (EAT) of high speed railway can be referred to as the following formula (9):

(9) $$\begin{array}{c} \text{EAT}=\frac{A}{D}, \end{array}$$

where *A* is the maximum lateral acceleration threshold ensuring that the normal operation of the train can withstand without orbit (Gal), *D* is the maximum dynamic response coefficient of various structures of railway under different seismic wave excitation, and suggestive value is 2.55.

Researches show that when

 case *A* ≥ 120 Gal, the train begins to pour;
 case *A* ≥ 240 Gal, the train will completely overturn.

Therefore, we define *A* = 240 Gal and *A* = 120 Gal as the threshold of strong impact and general impact on the safe operation of the high speed railway train. And the earthquake magnitude threshold of high speed railway operation is calculated by different value method, which is shown in Table 3.

#### 2.2.3. Threshold under Rain Influence

Domestic railway department limits the train running speed based on the size of the rain.

If the rain runs moderately which lasts 12 (or 24) hours and the rainfall capacity arrives at 10.0 mm–22.9 mm (17 mm–37.9 mm), its speed should be reduced.

If the rain runs in a heavy rainy day which lasts 12 (or 24) hours, and the rainfall capacity reaches 23.0 mm–49.9 mm (33.0 mm–74.9 mm), the railway lines are supposed to be blocked and the train operation is supposed to be prohibited.

For the sake of dimensional consistency, we can turn the hour rainfall volume into annual rainfall volume by the following method: it is universal knowledge that our country's rain season will experience a period of 3 months that can be calculated by 12 rainfall times; thus, we categorize the annual rainfall volume into 900 mm, 1980 mm, and 2970 mm, respectively, as the moderate rainfall city, heavy rainfall city, and the storm rainfall city. Accordingly, we can calculate rainfall threshold effects on high speed railway compared with the provisions of the railway departments in Table 4.

#### 2.2.4. Other Environmental Factors Threshold

The current theoretical researches both at home and abroad pay less attention to the lightning, snowing, temperature, and snowfall which will definitely bring some influences on the characteristics of the high speed railway operations. Because it is difficult to set up a uniform standard to measure the factors, experts suggest that the reference value and the method of combining qualitative analysis can be employed to determine what degree of lightning, snow, and temperature influencing the high speed rail threshold. The environment impact assessment index of high speed railway can be discriminated as in Table 5.

## 3. High Speed Railway Environmental Impacts Attribute Recognition Model

Attribute recognition model is in essence the problems of multidimensional space between sample and attribution, which is proposed by professor Cheng and has been widely used in evaluation and classification. The sample space *X* = {*x*_1_, *x*_2_, *x*_3_,…, *x*_31_} has been calculated in 31 provinces and autonomous regions in our country, among which each has been given nine high speed rail environmental impact indexes as *I*_*j*_ (*j* = 1,2,…, 9), and the *j*th environmental impact assessment index value in the *i*th region is expressed as *x*_*ij*_ (*i* = 1,2,…, 31; *j* = 1,2,…, 9). *F* is defined on a sample space *X* ordered split sets, where the environmental impact is divided into five progressive ways as serious, severe, moderate, mild model, and no effect. An ordered set of split is defined as *F* = {*C*_1_, *C*_2_, *C*_3_, *C*_4_, *C*_5_}, which is in accordance with the relationship as *C*_1_≻*C*_2_≻*C*_3_≻*C*_4_≻*C*_5_. Each ordered set is then to be split into a collection of environmental evaluation threshold segmentation classes. To make a clear illustration of the ordered stripe set, a standard form has been set up as follows:

(10) $$\begin{array}{c} \begin{array}{c} \begin{array}{c} \begin{array}{c} \begin{array}{c} I_{1}\\ I_{2} \end{array}\\ \vdots \end{array}\\ I_{9} \end{array} \end{array}\begin{array}{c} \begin{array}{ccccc} C_{1} & C_{2} & C_{3} & C_{4} & C_{5} \end{array}\\ \left|\begin{array}{ccccc} a_{11} & a_{12} & a_{13} & a_{14} & a_{15}\\ a_{21} & a_{22} & a_{23} & a_{24} & a_{25}\\ \vdots & \vdots & \vdots & \vdots & \vdots \\ a_{91} & a_{92} & a_{93} & a_{94} & a_{95} \end{array}\right| \end{array}, \end{array}$$

where *a*_*ij*_  (*i* = 1,2,…, 9; *j* = 1,2, 3,4, 5):  *a*_*i*1_ > *a*_*i*2_ > *a*_*i*3_ > *a*_*i*4_ > *a*_*i*5_.

The value of the sample properties has attributes characterized by a sample *X*_*i*_ and expressed as *u*_*ik*_ = *u*  (*u*_*i*_ ∈ *C*_*k*_), among which the measurement function is the core of attribute recognition model. Hu et al., Yan, and Xiao et al. make an analysis of the usual linear discriminated function, whose accuracy is less than that of a nonlinear function. Therefore, the recent researches have found that the normal distribution function is used much more frequently, while other nonlinear functions are often being regarded as an attribute identification measure function [12–14]. However, the normal distribution function as a measure function has its shortcomings because data should be standardized before handling bias and the separated index weights should also be determined. What is more, the last attribute recognition result is relative.

However, there is no certain way to evaluate the relative importance of objective indicators in a fairly way. The essence of attribute recognition is to determine the attributes space similarity and methods used to calculate the spatial distance are Euclidean distance, Ming distance, and Mahalanobis distance. Todeschini et al. and Kayaalp and Arslan assert that the Mahalanobis distance has the advantages of weakening the correlation between impact indicators and automatic weight in the index calculation based on data changes [15, 16].

Therefore, in order to compensate for normal function, we use Mahalanobis distance as the measurement function to build the attribute recognition model.

**Step 1 (Mahalanobis distance between sample and attribute class calculations).**

Assuming the sample *X*_*i*_ has been an area of environment evaluation, the sample Mahalanobis distance with the attribute class *C*_*k*_ is

(11) $$\begin{array}{c} d_{ik}=\sqrt{\left(X_{i}-C_{k}\right)\Sigma_{ik}^{-1}{\left(X_{i}-C_{k}\right)}^{T}}, \end{array}$$

where *X*_*i*_ = (*x*_*i*1_, *x*_*i*2_,…, *x*_*i*9_), representing the *i*th region environment factor evaluation vector, and *C*_*k*_ = (*a*_*k*1_, *a*_*k*2_,…, *a*_*k*9_), representing each classification criteria value of environmental factors on the properties class *k* vector. Σ_*ik*_ = the covariance matrix between *X*_*i*_ and *C*_*k*_ is

(12) $$\begin{array}{c} \Sigma_{ik}=\left[\begin{array}{cccc} \text{Cov}\left(x_{i1},a_{k1}\right) & \text{Cov}\left(x_{i1},a_{k2}\right) & \cdots & \text{Cov}\left(x_{i1},a_{k9}\right)\\ \text{Cov}\left(x_{i2},a_{k1}\right) & \text{Cov}\left(x_{i2},a_{k2}\right) & \cdots & \text{Cov}\left(x_{i2},a_{k9}\right)\\ \cdots & \cdots & \cdots & \cdots \\ \text{Cov}\left(x_{i9},a_{k1}\right) & \text{Cov}\left(x_{i9},a_{k2}\right) & \cdots & \text{Cov}\left(x_{i9},a_{k9}\right) \end{array}\right], \end{array}$$

where Cov(*x*, *y*) = *E*[(*x* − *E*(*x*))(*y* − *E*(*y*))].

**Step 2 (standard attribute measurement value calculations).**

Generally, the greater the similarity of Mahalanobis distance, the smaller the measurement value. Therefore, assuming that Mahalanobis distance between area *X*_*i*_ and attribute class *C*_*k*_ has been derived *d*_*ik*_, the standard attribute measurement value is

(13) $$\begin{array}{c} u_{ik}=\frac{1/d_{ik}}{\sum_{j=1}^{5}\left(1/d_{ij}\right)}. \end{array}$$

**Step 3 (sample class attribute recognition).**

Class attribute identification is in accordance with the confidence value *λ*:

(14) If  ki=min⁡k:∑l=1kuil≥λ,k=5,4,3,2,1Then  Xi  Can  be  considered  as  class  Ck,

where *λ* normal circumstances take 0.6 ≤ *λ* ≤ 0.7.

**Step 4 (security score calculations).**

Assuming each evaluation category *C*_*k*_ corresponding score of *q*_*k*_, then the combined attribute security score is

(15) $$\begin{array}{c} S_{i}=\overset{4}{\underset{k=1}{\sum }}u_{ik}q_{k}. \end{array}$$

## 4. Case Studies

### 4.1. Chinese Regions Environment Overview

Five domestic environmental factors such as rainfall, lightning, wind, temperature, and earthquake in recent years are collected from 2002 to 2012 as the basic assessments data [17] as is shown in Table 6. (The data of rain factor is summary of annual average rainfall in various regions, the data of thunder and lightning factors comes from various regions' monitoring reports, and the data of wind factor represents the influence extent by monsoon in various regions.)

The program of MATLAB is employed to work out the estimation. The specific method is made by 31 districts samples and each has 9 indexes. Then we constitute the sample matrix *R*_31×9_. There are five characteristics consisting of particularly serious, severe, moderate, mild, and no effect, whose intermediate values will be made up of attribute matrix *R*_5×9_; that is,

(16) $$\begin{array}{c} R_{5\times 9}=\left[\begin{array}{ccccc} 35.0 & 32.5 & 27.5 & 22.5 & 10.0\\ 3.00 & 2.50 & 1.50 & 0.75 & 0.25\\ 30.0 & 26.0 & 19.5 & 13.0 & 4.50\\ 5.20 & 5.00 & 4.60 & 4.15 & 1.95\\ 1.00 & 0.80 & 0.45 & 0.20 & 0.05\\ 55.0 & 50.0 & 40.0 & 22.5 & 7.50\\ 2970 & 2475 & 1440 & 750 & 300\\ 64.0 & 56.0 & 41.5 & 30.0 & 17.5\\ -20.0 & -15.0 & -5.00 & 2.50 & 7.50 \end{array}\right]. \end{array}$$

Use the function pdist of MATLAB to work out the Mahalanobis distance between the districts sample and the attribute class:

(17) $$\begin{array}{c} z=\text{pdist}\left(R_{31\times 9},R_{5\times 9},\text{“mahal”}\right), \end{array}$$

where *z* is the Mahalanobis distance matrix between the sample and the attribute and mahal is representing the use of the function Mahalanobis distance to work out the distance of matrix.

Then make confidence level *λ* = 0.60, and each of the area's environmental attribute recognition values and attribute classification can be obtained as that in Table 7.

**Figure 2:**
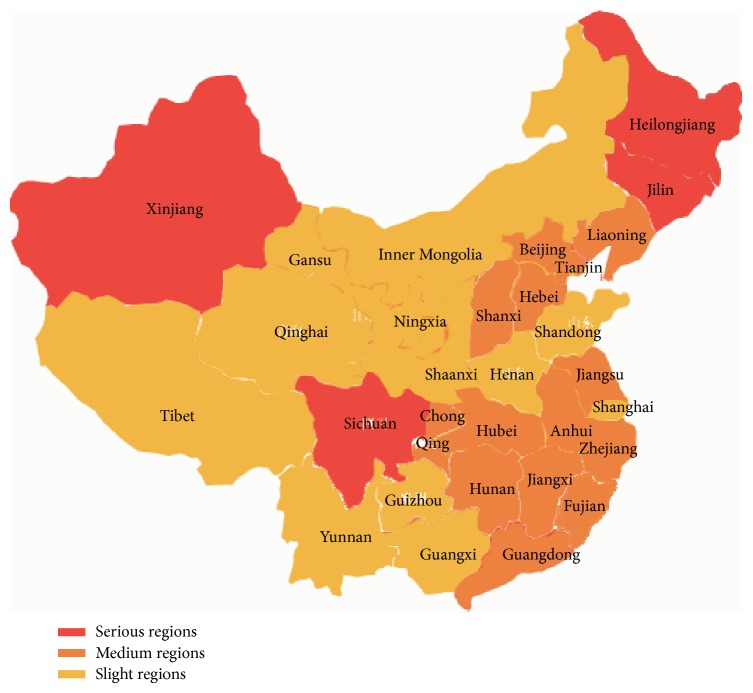
Chinese environment impacts of high speed railway distribution.

The calculation results in the above table show that the environmental safety situation of Xinjiang, Sichuan, Heilongjiang, and Jilin belongs to serious category, which takes up 12.9%. The situation in the Medium level areas accounts for 32.2%, such as Heilongjiang, Hebei, Liaoning, Jiangsu, and Guangdong, and that of the 17 areas such as Beijing, Tianjin, Guizhou, Gansu, and other regions belongs to slight level, which accounts for 54.9%. It is notable that, in addition to Sichuan, the high speed railway environment impacts in the serious level areas are mostly distributed in coastal areas and northern regions, while Chinese abdominal regions are mostly in the medium and light level (see Figure 2).

For further analysis, security score in the areas of the serious level should be calculated by means of grading criterion. All kinds of scores are clarified in Table 8.

The calculation results show that Sichuan has the lowest scores of 62.460, followed by 63.280 in Heilongjiang and 63.489 in Xinxiang, and Yunnan has the highest score of 72.23.

### 4.2. High Speed Railway Line Safety Environment Analysis

There are 25 high speed railway operational lines in our country currently, which constitute the total mileage of 10192 kilometers. Most of the high speed railways are located in southeast of China, where complex geological accidents such as landslip, earthquake, and other geological disasters take place frequently. The high speed railway environment safety situation is clearly illustrated in Table 9.

The Jinghu line, Fuxia line, and Huning line mainly go across regions of Beijing, Tianjin, Jinan, Nanjing, Shanghai, Hangzhou, and so on. Most of these regions are located in the medium impacted or light impacted areas where raining and storm happen frequently. Thus, we have to pay attention to the influence of heavy rain and storm.

The Wuguang line and Guangshengang line mainly go cross such cities as Guangzhou, Foshan, and others in Guangzhou. These cities are vulnerable to the typhoon from coastal regions, which will affect the progress of the high speed railway.

The Yiwan line, Suiyu line, and Dacheng line go cross Wanzhou, Suining, Shizishan, Chengdu, or other cities of Sichuan province. High speed railway in these areas will suffer seriously from the tough environment, and we should pay attention to prevent cost and loss from landslip and earthquake.

## 5. Conclusions

Firstly, the paper makes a detailed analysis of the impact from such environment factors as rainfall, earthquake, lightning, wind, and snow on the high speed railway safety mechanism. On the basis of the analysis, the evaluation index system of safety has been established and the threshold of high speed railway environmental safety has been calibrated by citing the results of domestic and abroad. At last, the high speed railway uncertain safety attribute recognition model is created based on the Mahalanobis distance with the features of dimensionless and weak effect correlation, which simplifies the comprehensive calculation process.

Secondly, the examples of China's 31 provinces and regions in the paper are selected to make the data of the high speed railway environmental safety much more convincing. The degree of danger is divided into five categories, among which the cities that the high speed railways pass in the serious category account for 16.1%, those in the middle class account for 38.7%, those in the mild category account for 38.7%, and those in the no effect category account for 6.51%. It deserves our attention that cities of Xinjiang, Sichuan, Guangdong, Heilongjiang, and Liaoning belong to the serious category, whose evaluation results are basically consistent with the environmental characteristics. And the results have a certain theoretical reference for the “135” planning of high speed railway operation safety in Xinjiang and other areas.

At last, the analysis of the high speed railway environmental safety is directed to the aspect of weather, geology, and other factors. However, considering the complexity of data acquisition, the high speed railway evaluation index has its own drawbacks in this paper. It is needed to introduce more methods and factors into the evaluation of the high speed railway safety operation to facilitate the further researches.

## Figures and Tables

**Table 1:** High speed railway mechanism analysis of environmental impact factors.

Environmental factor	Mechanism
Rainfall	(i) Raining is the foremost factor that is easily causing line fault. Additionally, the current flow will emerge between the pantograph and overhead line systems of the high speed railway when it comes to a heavy rainy day and the train power supply will also be consequently influenced. (ii) Rainfall can cause mountain soil landslides that will directly lead to the abnormal operation of the train.

Cross wind	(i) The mechanism of the influence caused by horizontal wind on the high speed railway is that it can produce the yawing force which will allow the lateral migration. Moreover, the produced lift force will lead to the train derailment through the pneumatic action with the high speed train, which will undeniably increase the risk of train being derailed.

Lightning	(i) Lightning can disrupt the power supply of the high speed railway train traction which will result in the sudden stop of the high speed rail train through breaking down the high speed railway along the circuit devices.

Earthquake	(i) Earthquake wave can be divided into two kinds: the P wave (primary wave, pressure wave) and the S wave (secondary wave, shear wave). S wave can destroy the building structure and cause the landslides, orbital shift, and train wheel derailment which will influence the safe driving of high speed railway.

Temperature	(i) High temperature can lead to a big temperature difference between the internal and external, the increase of air conditioning power, and the aggravation of the train power supply load. Besides, high temperature can cause the short circuit because of the softened line. (ii) Cold damp climate will lead to the ice covering membrane on the railway track, which will reduce the friction between train and track and increase the risk of a derailment when the train is at high speed turning.

Snowfall	(i) A lot of snow will cover the track and the ice on the track will increase the degree of danger of the train operation.

**Table 2:** Japanese Shinkansen winds threshold.

Wind scale	Wind speed (m/s)	The impact with no wind-break wall	The impact with wind-break wall
8.0-9.0	20–25	The train speed under 160 km/h	No speed limit
9.0–10.4	25–30	The train speed under 70 km/h	The train speed under 160 km/h
10.4–12.5	30–35	Off-stream	The train speed under 70 km/h
Above 12.5	Above 35	Off-stream	Off-stream

**Table 3:** Earthquake magnitude threshold of high speed railway (*D* takes 2.55).

Rank	Very serious	Serious	General	Slight	No effect
Train lateral acceleration (*A*)	240 Gal	180 Gal	120 Gal	60 Gal	0 Gal
Earthquake magnitude (EAT)	>5.2	4.8	4.4	3.9	<3.9

**Table 4:** The annual rainfall threshold of high speed railway.

Rank	Very serious	Serious	General	Slight	No effect
Annual rainfall	>2970 mm	1980 mm	900 mm	600 mm	<600 mm

**Table 5:** High speed railway environment impact assessment index discrimination safety threshold.

Environment	Evaluation Index	Particularly serious	Serious	Medium	Slight	No effect
		*C*_1_	*C*_2_	*C*_3_	*C*_4_	*C*_5_
Cross wind	*X*_1_ (m/s)	>30	25–30	15–25	5–15	0–5
	*X*_2_ (time)	>3	2-3	1-2	0.5–1.0	0–0.5
Snowfall	*X*_3_ (cm)	>30	22–30	17–22	9–17	0–9
Earthquake	*X*_4_ (level)	>5.2	4.8–5.2	4.4–4.8	3.9–4.4	0–3.9
	*X*_5_ (time)	>1.0	0.6–1.0	0.3–0.6	0.1–0.3	0-0.1
Lightning	*X*_6_ (time/km^2^)	>55	45–55	30–45	15–30	0–15
Rainfall	*X*_7_ (mm)	>2970	1980–2970	900–1980	600–900	0–600
Temperature	*X*_8_ (°C)	>64	48–64	35–48	25–35	10–25
	*X*_9_ (°C)	<−20	−10–−20	−10–0	0–5	5–10

*X*_1_: average disaster annual wind speed.

*X*_2_ **: **the annual incidence of disasters monsoon.

*X*_3_: annual maximum snow depth.

*X*_4_: the average magnitude level.

*X*_5_: average annual rate of earthquake occurrence.

*X*_6_: annual lightning density.

*X*_7_: annual maximum rainfall.

*X*_8_: average annual maximum temperature.

*X*_9_: average annual minimum temperature.

**Table 6:** Chinese regional environment situation in recent years from 2002 to 2012.

Region	Index								
	*X*_1_	*X*_2_	*X*_3_	*X*_4_	*X*_5_	*X*_6_	*X*_7_	*X*_8_	*X*_9_
Beijing	0	0	22.5	0	0	13.61	498.96	26.86	−2.75
Tianjin	41.67	0.11	19.7	0	0	8.6	504.6	26.84	−3.71
Hebei	47.92	0.22	20	7.8	0.03	29.97	544.97	27.48	−1.77
Shanxi	0	0	21	0	0	27.41	443.46	24.48	−5.11
Inner Mongolia	0	0	17	0	0	0.6	373.29	23.56	−10.87
Liaoning	41.67	0.11	25	7.3	0.02	8.75	701.63	24.3	−12.01
Jilin	26.39	0.11	27	0	0	9.25	598.41	23.38	−14.6
Heilongjiang	0	0	34	0	0	15.44	498.9	23.24	−16.9
Shanghai	32.99	0.89	8	0	0	17.176	1092.41	29.39	4.75
Jiangsu	35.14	1.11	22	0	0	40.25	1164.72	28.81	2.95
Zhejiang	39.22	2.78	6	0	0	76	1276.82	29.95	4.84
Anhui	36.87	1.22	15	0	0	35.75	1057.21	28.74	2.76
Fujian	39.56	3.22	4	0	0	35.3	1355.53	29.81	11.3
Jiangxi	38.8	1.78	12	0	0	35	1500.14	30.13	5.53
Shandong	33.8	0.33	17	0	0	32.5	820.57	26.98	−1.21
Henan	42.13	0.33	19	0	0	30.67	724.81	27.29	1.4
Hubei	40.67	0.78	17	0	0	26.72	1210.48	29.83	4.38
Hunan	35.52	0.78	16.4	0	0	29	1276.44	29.96	8.39
Guangdong	33.93	4.11	0	0	0	48.25	1805.49	29.78	13.16
Guangxi	34.92	2.33	4	0	0	26.25	1189.73	28.3	14.49
Hainan	31.74	2.22	0	0	0	38.75	1780.62	29.06	14.07
Chongqing	0	0	3.7	0	0	23.58	1065.61	29.53	6.89
Sichuan	0	0	4.2	7.44	0.1	57.256	843.16	25.96	5.98
Guizhou	43.06	0.11	4.5	0	0	31.75	989.78	23.19	3.52
Yunnan	35.19	0.33	0	7.33	0.1	27.21	878.28	21	9.62
Tibet	0	0	52	0	0	0.29	453.12	17.31	0.62
Shanxi	15	2	19	0	0	15.46	611.11	27.61	0.36
Gansu	12.4	2.22	18	6.6	0.02	0.36	271.74	22.46	−5.06
Qinghai	32	3.56	15	6.9	0.02	0.42	442.9	17.48	−7.75
Ningxia	4.72	1.89	17.9	0	0	4.77	175.34	24.31	−7.38
Xinjiang	46	4.67	46	7.1	0.05	0.25	309.61	24.19	−12.9

**Table 7:** Chinese regional environment impacts attribute recognition value of high speed railway.

Region	Value					
	Particularly serious	Serious	Medium	Slight	No effect	Classification
Xinjiang	0.301	0.402	0.117	0.003	0.177	Serious
Sichuan	0.376	0.246	0.196	0.120	0.062	
Jilin	0.303	0.363	0.146	0.082	0.106	
Heilongjiang	0.269	0.342	0.215	0.140	0.034	

Hebei	0.169	0.196	0.237	0.231	0.168	Medium
Liaoning	0.201	0.203	0.218	0.198	0.180	
Jiangsu	0.177	0.209	0.228	0.211	0.174	
Zhejiang	0.180	0.202	0.225	0.205	0.188	
Anhui	0.166	0.202	0.234	0.221	0.177	
Jiangxi	0.196	0.222	0.206	0.199	0.178	
Hubei	0.179	0.210	0.221	0.216	0.175	
Hunan	0.175	0.205	0.221	0.222	0.176	
Guangdong	0.195	0.200	0.212	0.198	0.196	
Fujian	0.194	0.211	0.195	0.200	0.200	

Beijing	0.163	0.193	0.226	0.239	0.179	Slight
Tianjin	0.158	0.188	0.232	0.234	0.187	
Shanxi	0.160	0.190	0.227	0.228	0.195	
Inner Mongolia	0.178	0.200	0.202	0.212	0.208	
Shanghai	0.173	0.203	0.215	0.224	0.185	
Hainan	0.168	0.198	0.222	0.224	0.189	
Shandong	0.162	0.196	0.234	0.222	0.186	
Henan	0.150	0.184	0.247	0.237	0.182	
Guangxi	0.151	0.181	0.222	0.248	0.199	
Chongqing	0.180	0.201	0.208	0.223	0.187	
Guizhou	0.162	0.187	0.202	0.206	0.244	
Yunnan	0.153	0.177	0.201	0.229	0.239	
Tibet	0.178	0.192	0.202	0.213	0.215	
Shaanxi	0.155	0.187	0.235	0.245	0.178	
Gansu	0.165	0.191	0.215	0.237	0.192	
Qinghai	0.177	0.194	0.194	0.203	0.231	
Ningxia	0.155	0.183	0.224	0.237	0.200	

**Table 8:** The attribute recognition of high speed railway classification score.

Classification	No effect	Slight	Medium	Serious	Particularly serious
Score	90	80	70	60	50

**Table 9:** The environment impacts of high speed railway lines distribution.

Lines	Serious environmental impact	Middle environment impact	Light environmental impact
Jinhu	—	Nanjing, Jinan	Beijing, Tianjin, and Shanghai
Hebang	—	Hefei, Bengbu	—
Jinshi	—	Shijiazhuang	Beijing
Wuguang	—	Wuhan, Changsha	Guangzhou, Foshan
Guangshengang	—	—	Guangzhou, Shenzhen
Yongtaiwen	—	—	Ningbo, Taizhou, and Wenzhou
Wenfu	—	Wenzhou, Fuzhou	—
Fuxia	—	Fuzhou, Xiamen, and Quanzhou	—
Zhenxi		—	Zhengzhou, Xi 'an
Xibao	—	—	Xi 'an, Baoji
Huhang	—	Jiaxing, Hangzhou	Shanghai
Hewu	—	Nanjing, Hefei, and Wuhan	—
Hanyi	—	Hankou, Zhijiang, and Yinchuan	—
Hening	—	Hefei, Nanjing	—
Jiaoji	—	—	Jinan, Qingdao
Shitai	—	—	Shijiazhuang, Taiyuan
Huning	—	Nanjing, Suzhou	Shanghai
Yiwan	Wangzhou, Ensi	—	Yichang
Yuli	—	Chongqing, Fuling	Liangwu
Suiyu	Suining, Shizuishan	—	—
Dacheng	Dazhou, Suining, and Chengdu	—	—
